# Is the Correlation between Storage Capacity and Matrix Reasoning Driven by the Storage of Partial Solutions? A Pilot Study of an Experimental Approach

**DOI:** 10.3390/jintelligence5020021

**Published:** 2017-05-16

**Authors:** Florian Domnick, Hubert D. Zimmer, Nicolas Becker, Frank M. Spinath

**Affiliations:** 1 Individual Differences & Psychodiagnostics, Department of Psychology, Saarland University, Building A1 3, 66123 Saarbrücken, Germany; nicolas.becker@mx.uni-saarland.de (N.B.); f.spinath@mx.uni-saarland.de (F.M.S.); 2 Brain & Cognition Unit, Department of Psychology, Saarland University, Building A2 4, 66123 Saarbrücken, Germany; huzimmer@mx.uni-saarland.de

**Keywords:** fluid intelligence, matrices test, working memory, storage capacity, partial solutions

## Abstract

Working memory capacity (WMC) and reasoning abilities—as assessed by figural matrices tests—are substantially correlated. It is controversially discussed whether this correlation is only caused by controlled attention or also by storage capacity. This study aims at investigating storage of partial solutions as a possible mechanism by which storage capacity may contribute to solving figural matrices tests. For this purpose, we analyzed how an experimental manipulation of storage demands changes the pattern of correlations between WMC and performance in a matrix task. We manipulated the storage demands by applying two test formats: one providing the externalization of partial solutions and one without the possibility of externalization. Storage capacity was assessed by different types of change detection tasks. We found substantial correlations between storage capacity and matrices test performance, but they were of comparable size for both test formats. We take this as evidence that the necessity to store partial solutions is not the limiting factor which causes the association between storage capacity and matrices test. It is discussed how this approach can be used to investigate alternative mechanisms by that storage may influence performance in matrices tests.

## 1. Introduction

In this study, we experimentally manipulated storage demands of a figural matrices test and observed its relationship to storage capacity as one definition of working memory capacity (WMC). Although working memory capacity plays a fundamental role in fluid cognitive abilities (gF), mainly assessed with figural matrices tests (e.g., [1,2,3,4,5,6,7,8]), the reasons for this interrelationship are not well understood, and the explanation of this effect depends on the theoretical perspective and definition of WMC. Two definitions of WMC are commonly discussed: WMC as the result of controlled attention [9] or WMC as a fixed amount of available storage space [10]. According to the first perspective, attention is decisive for the amount of information that can be held in working memory. Storage per se is not the limiting factor but a consequence of the efficiency of attentional control. Based on this assumptions, retaining relevant information in the face of distraction and interference are supposed to be critical functions of the working memory system associated with gF (e.g., [1,2,8]).

**Figure 1:**
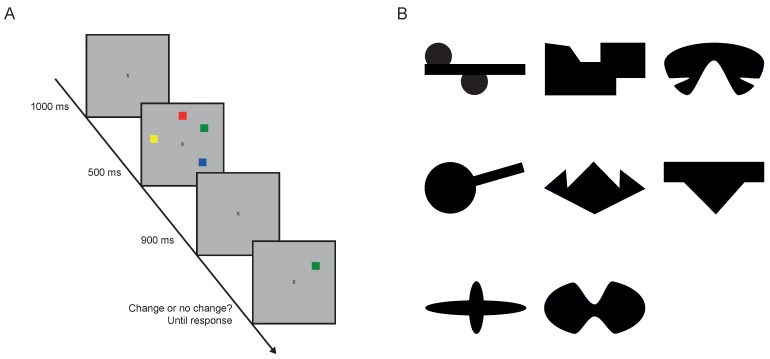
(**A**) Illustration of the task procedure at the example of the color-change task; (**B**) The difficult to be named shapes used in the shape-change taks.

In contrast, according to the second perspective, storage per se is limited. It is assumed that only a small number of three to four items can be stored within working memory why such models are sometimes called slot models [11]. WMC is often termed as storage capacity [10,12] in this context and is estimated by the *change detection paradigm* [13]. In a change detection task (see Figure 1A for an example) a sample of items is briefly shown for a few hundred milliseconds and re-presented after a short delay. The re-presented sample is identical or differs in some aspect (e.g., color or shape is changed). Individuals are requested to judge if the display is the same as in the first presentation or not. By varying the number of displayed objects, individuals’ storage capacity as the number of simultaneously retained items can be estimated. A major advantage of this measure is that strategical techniques such as rehearsal are minimized, and therefore, the paradigm allows for a less biased measurement of storage capacity. Research regarding the impact of storage capacity on gF demonstrates that storage capacity is highly predictive of gF [12,14,15]. However, it remains unclear what drives this relation and how exactly storage capacity could be connected to gF tasks, especially to figural matrices tasks. In this study, we want to shed light on the possible role of storage capacity in storing partial solutions as one critical storage component in matrices tests. To this end, we experimentally manipulated the storage of partial solutions in figural matrices test and explored if storage capacity has an impact on this specific process. We will briefly review a framework of a well-established theory for solving behavior in figural matrices along with potential influences of WMC on figural matrices tests.

## 2. Figural Matrices and Storage Demands

Studies investigating the relationship between working memory and gF typically utilize figural matrices tasks to assess gF [16,17,18,19]. Although one task cannot fully represent an underlying construct, figural matrices can be regarded as core tasks for gF and problem solving and are preferentially used for estimating individuals’ gF [20]. Additionally, solution processes can be studied using experimental designs [16,17,21]. Consequently, certain sub-processes in solving behavior in figural matrices tests can be observed and experimentally manipulated to study insights of WMC influences on gF tasks.

### 2.1. Processes in Figural Matrices

A typical figural matrices task contains a 3×3 matrix filled with patterns that follow specific design rules. The lower right field (solution field) is usually left empty, and the solver has to fill the field according to the applied rules. Traditionally, the correct answer (attractor) is presented along with response alternatives (distractors). The solver has to determine which response option is the attractor. Using verbal reports, eye-tracking and computational simulations, Carpenter et al. [22] described the solution process in Raven’s Advanced Progressive Matrices (APM) [23], a common example of figural matrices tests. They distinguish three main processes: perceptual analysis, conceptual analysis, and response generation. While the perceptual analysis is responsible for finding correspondences between related sub-patterns, the conceptual analysis enables an induction and validation of rules. After all rules are applied to the whole matrix, the final solution can be selected from eight response alternatives. Since visual representations of the patterns and its correspondences to applied rules have to be stored, Carpenter et al. [22] accentuate the need for a working memory module that could act like a sketchpad or an intermediate storage. As figural matrices usually involve more than one rule, the final solution consists of several sub-results or partial solutions which have to be additionally stored within working memory until the whole item is solved and the answer can be generated. For successful matrix solving, a superordinate goal monitor manages stored visual information of the matrix and creates sub-goals to keep track of the aim of the current task. The sub-goals contain sequences of solution steps that individuals have to follow to solve an item successfully. Especially, serial processing of sub-goals and focusing on relevant task goals are the main roles of the goal monitor.

### 2.2. The Role of WM in Figural Matrices

The model of Carpenter et al. [22] posits the crucial role of working memory in figural matrices tests. Particularly, executive processes such as goal monitoring and serial processing of sub-goals are core parts of the model. This is in line with latent variable approaches highlighting the role of executive functions of working memory in gF [1,2,8]. Using complex span tasks, Engle et al. [1] found that the main aspect driving the association of working memory and gF was controlled attention. Engle [9] describes that “capacity is not about individual differences in how many items can be stored per se but about differences in the ability to control attention to maintain information in an active, quickly retrievable state” (p. 20). Consequently, individuals with high WMC should perform better because they can maintain and manipulate relevant information in the face of interference and distraction more efficiently. For example, Jarosz and Wiley [16] found that individuals with low WMC were more negatively affected by a salient distractor in a figural matrix item than those with high WMC. Wiley et al. [24] found that correlations between WM and matrix reasoning scores increased when new rules were applied in figural matrices tasks which they explained by proactive interference. Old rules from previous items are still retained in memory and individuals with low WMC are unable to inhibit these solutions principles when performing the matrix with new rules. However, other studies have report the opposite result, in which memory of repeated rules was advantageous [18,21]. Loesche et al. [17] demonstrated that the correlation between WMC and APM performance remained the same or even increased when all rules were known in advance so that performance was mainly limited by action monitoring and memory of sub-goals.

In summary, these studies demonstrate that WMC defined as controlled attention is necessary for matrix reasoning to inhibit (proactive) interference, successfully retrieve rule principles over items and managing sub-goals within an item with multiple rules. Hence, WMC in this context is responsible for preventing that the respondents’ attention is drawn into distraction and enables that the respondent is keeping track of the current task goals. However, there are indications that not only the ability to control attention within the task but also the amount of information which can be stored within working memory is crucial for successfully solving the matrix. Results of latent-variable approaches indicate that WMC defined as storage capacity shares unique variance with gF besides controlled attention [12,25,26,27,28]. Storage capacity is regarded as the number of objects that can be stored in an accessible state within the focus of attention [10]. The size of this region, i.e., the number of accessible objects, is highly limited and varies between individuals [15,29]. In terms of gF, the assumption is that higher storage capacity enables to attend to more information in a task. According to Unsworth and colleagues [25] this means “that high capacity individuals can simultaneously attend to multiple goals, sub-goals, hypotheses, and partial solutions for problems which they are working on allowing them to solve better the problem than low-capacity individuals who cannot maintain/store as much information. (p. 3)”. In fact, there is evidence that especially the storage of partial solutions is crucial for higher order cognition, such as mental arithmetic [30] or reading comprehension [31]. The question is, how this conclusion can be transmitted to matrix reasoning. As pointed out in the previous section, according to the model by Carpenter et al. [22], WMC is required in matrix reasoning to store and access current representations, sub-goals and intermediate stages of the solution, which includes partial solutions. Thus, WMC in matrix reasoning can be regarded as the “size” of a sketchpad that helps to store relevant information about the current problem and performance could depend on individual storage capacity (i.e., how much information can potentially be stored within working memory) of the respondent. Especially, figural matrices items with multiple rules require a storage of partial solutions since the final solution is consisting of the results of each rule, and the partial solutions have to be maintained within working memory until the whole item is solved.

## 3. The Present Study

To address if a sketchpad in figural matrices tests is necessary to store partial solutions, and if this is related to individuals’ storage capacity, we experimentally manipulated storage demands of partial solutions in one figural matrices test. We tested individuals’ matrix reasoning ability with two versions: the first version provides a sketchpad that enables externalization of partial solutions and the second version requires the storage of partial solutions until the whole item is solved. We hypothesized that enabling externalization of partial solutions to a sketchpad relieves the working memory module in the matrices tests. Hence, if storage of partial solutions is necessary for successfully solving figural matrices tests, performance should be better when externalization is provided compared to the condition in which externalization is prevented. Importantly, if storage capacity is involved in storing partial solutions, variability in storage capacity should explain more variance in the matrices test in which partial solutions have to be retained compared to the version where partial solutions can be externalized. In other words, the correlation between storage capacity and matrices test performance should be stronger in the non-externalized condition compared to the externalized condition.

Our aim was to experimentally manipulate storage demands in figural matrices tests while controlling for other item characteristics that can influence the solving behavior in figural matrices tests. For instance, there is a large body of research that has shown that type of rule [22,32] or the number of rules in an item [32,33,34,35,36,37] can affect the solving process in figural matrices test, which could alter the contributions of working memory. Therefore, we applied an adaption of the ”Design a Matrix” test (DESIGMA) [34,38,39] that enabled to control for these influences. First, the type of rule was counterbalances in matrices tests items in both conditions. Second, although in traditional progressive matrices tests (e.g. APM [23]) the number of rules is increasing from simple to complex items, we kept the number of rules constant. The number of rules could affect the (visual) complexity in an item, and other processes could be demanded besides storage of partial solutions such as selective encoding or goal management (e.g., [33,35]).

## 4. Method

### 4.1. Participants and Design

Eighty-five students from Saarland University were tested and received monetary compensation (15 €). Due to missing data, one participant had to be excluded from further analyses. The sample (56 female) had a mean age of 23.3 (SD = 3.8, range 18–35) years.

Participants were assessed in group settings on individual computers with ear protection in order to avoid distraction. The sessions did not exceed two hours. For stimulus presentation and data recording, PsychoPy 1.81.03 [40] was used.

We applied a within-subject design, in which every participant worked on the matrices tasks in both conditions. Additionally, we assessed storage capacity with three types of change detection tasks. Both the order of the three change detection tasks and the order of the two matrices tests were counterbalanced across participants. Since a within-design was applied, two different item sets for matrices test were designed. Each matrices test contained one of these two sets. In order to rule out confounding factors of specific items on one matrices test, the assignment of item sets to matrices tests was also counterbalanced across participants.

### 4.2. Test Methods

#### 4.2.1. Change Detection

We measured the individual working memory capacity with three different blocks of change detection tasks with a change of color, shape, and orientation [13]. In order to apply a design comparable to Fukuda et al. [15], we operationalized the change detection task with big change and single probe presentation at test-display (see Figure 1A). First, a sample array with four or six randomly chosen objects was presented for 500 ms. After a blank screen of 900 ms, a test display with only one object appeared at a random position until a response was given. Participants had to detect if the objects’ relevant feature (color, shape, orientation) at this position had changed. In 50% of the trials, the test object was identical to the object at the same location in the sample display whereas for change conditions a randomly chosen object that had not been shown within the sample display was presented. Importantly, in the change-conditions, only the critical feature was changing (e.g., color) and the irrelevant was fixed (e.g., shape of colored squares). Participants were instructed in advance which feature is potentially changing.

We used eight different colors (green, blue, red, yellow, white, black, violet, cyan), eight shapes (see Figure 1B) and eight orientations ($0^{\circ }$, $45^{\circ }$, $90^{\circ }$, $135^{\circ }$, $180^{\circ }$, $225^{\circ }$, $270^{\circ }$, $315^{\circ }$) of the letter T as stimuli. For each block (color, shape, orientation) 40 trials with set size 4 and 40 trials with set size 6 were presented. Additionally, seven practice trials were shown before each block. All stimuli were presented within the $9.53^{\circ }$ × $9.53^{\circ }$ region on the monitor and were separated by at least $3^{\circ }$ (center to center). All stimuli were presented on an invisible circle around the fixation cross with a radius of $3.92^{\circ }$.

#### 4.2.2. Matrices Tests

**Figure 2:**
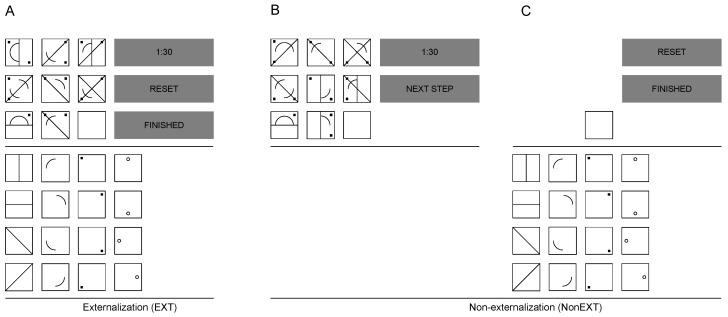
Illustration of the two versions of matrices tests (Ext and NonExt). (**A**) Ext with possibility of immediate response; (**B**) Inducing step of NonExt. No response can be given; (**C**) Responding step of NonExt. The matrix disappears directly after confirming *next step* in (B). For better understanding, control button descriptions were translated from German to English for this article.

Two modified versions of the “Design a Matrix” test (DESIGMA) [34,38,39] were used. The first version (Ext) allows externalization as the opportunity of immediate response while induction was given, whereas the second version (NonExt) forces subjects to memorize each items’ full solution before responding (see Figure 2). Each item of Ext consisted of a 3 × 3 matrix with an empty cell (response field) in the right lower corner (see Figure 2A). Contrary to traditional matrices tests such as Raven’s APM, no set of response alternatives including the correct solution was presented. Instead, participants had to construct the solution using 16 elements given in a box below the matrix (construction field). Importantly, these 16 elements were the same elements every matrix was based on. After selecting one element from the construction field, the element appeared within the response field; choosing the same element for the second time deleted this element from the response field. A time limit of 90 s was given for each item, which was empirically determined (see [34]). The time remaining to enter the solution was permanently displayed in the upper-right corner of the screen. Additionally, the reset button offered the opportunity to clear all elements in the response field. Participants were instructed to click on the finish button when they believed that they had constructed the correct solution. After 90 s, the item terminated automatically. An optional break of 30 s was given before the next item was shown. Similar to Ext, items of NonExt were presented in a 3 × 3 matrix with an empty response field. In contrast to Ext, the construction field in NonExt remained invisible until the participant confirmed the button next step. Thus, participants had to solve the whole matrix mentally before responding and also had to memorize the item’s correct solution (inducing step, see Figure 2B). After confirming next step, the matrix disappeared, the construction field appeared, and the participants had to choose the elements from the construction field based on their mental representation (responding step, see Figure 2C). The solving time within inducing step was 90 s for each item. After 90 s or if participants confirmed next step, the responding step started. After the responding step (self-paced), an optional break of 30 s was given before the next item started. Instructions for Ext and NonExt were held constant, except for the instruction to respond immediately in Ext and to memorize the solution in NonExt before responding. To familiarize the participants with the testing procedure, one practice item was presented before each version of the matrices test. Both versions of matrices tests consisted of 16 randomly presented items. As pointed out in Section 4.1, two sets of structurally similar items were constructed and were put in either set A or set B.

To hold memory demands based on the number of given visual information during induction constant, each item consisted of three different rules. Combinations of rules and elements were counterbalanced across items. In total, we used four different rules: addition, subtraction, overlap, and addition of unique elements. Rules were applied over rows. Table 1 gives a short description of the main feature for each rule type. Rules were applied to four possible element groups: lines, circle segments, small squares, and small circles. These elements were exactly the elements shown in the construction field (see Figure 2A), ensuring that every item could be solved with elements of the construction field. Within each item, one rule was only applied to one element group (e.g., addition was applied to lines but not to lines and circles in Item X) so that no rule was repeated within an item.

For instance, in Figure 2A three rules were applied row-wise to the item: (1) the line elements from the first two cells are summed up in the third cell; (2) the circle elements from the second cell are subtracted from the first cell, and the result appears in the third cell; and (3) the little black squares that are shown in the first or the second cell (but not in both) appear in the third cell.

### 4.3. Data Analysis

#### 4.3.1. Change Detection

We estimated memory capacity as *k* which is the number of items that an individual can store in working memory. The estimation is based on the assumption that the participant gives a valid response of each item which is in memory and otherwise the answer is guessed. Hence, the proportion correct is corrected for guessing, and therefore, it is an estimate of the proportion of items which were really stored in working memory. We used the standard *k*-score formula k=setsize×(hitrate−falsealarmrate) by Cowan [10] to estimate the individual storage capacity. *k* represents the number of stored items in memory, setsize the number of presented objects in the sample display, hitrate the proportion of correctly detected changes, and falsealarmrate the proportion of given change responses to non-change trials. We calculated *k* for each condition and set size individually and used the average of *k* for set sizes 4 and 6 of each condition for further analyses. Higher values represent higher storage capacity. For correlation analyses, we calculated a joint storage capacity score using the average of *z*-values based on all storage capacity estimates (color, shape, orientation).

#### 4.3.2. Matrices Tests

We used the number of solved rules instead of the number of solved items for each matrices test version as an estimate for participants’ matrix reasoning ability. This gave us the opportunity to directly observe how many details of the solution were retained for an item. Higher values represent more solved rules. For structural equation modeling, we used item parcels, each consisting of four items that were summed up, resulting in four parcels which served as indicators for the latent cognitive ability factor.

#### 4.3.3. Statistical Analyses

Structural equation models and confirmatory factor analysis were conducted using lavaan 0.5–20 [41] with maximum likelihood as the estimator. The following conventions were used to assess the global fit of the model: RMSEA < .06, SRMR < .09 and CFI close to .95 [42].

## 5. Results

Descriptive statistics and correlations between gF and storage capacity estimates are presented in Table 2. Both matrices test versions were strongly correlated (*r* = .81, *p* < .001). However, significantly more rules were solved in Ext compared to NonExt (*t*(83) = 2.54, *p* < .05, *d* = 0.28). Please note that analyses based on the number of solved *items* revealed a similar result (*t*(83) = 2.53, *p* < .05, *d* = 0.28). Correlations of single storage capacity tasks with gF ranged from *r* = .13 to .35 for Ext and from *r* = .11 to .27 for NonExt. Importantly, correlations of Ext and NonExt with storage capacity (Mean *k*) were equivalent (*t*(84) < 1).

**Figure 3:**
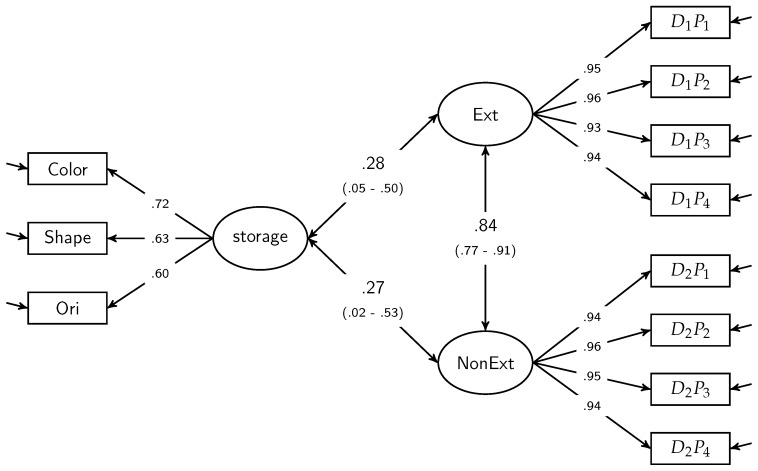
SEM for interrelations between storage capacity and the two versions of matrices tests applied in this study (Model 1). Parameters are standardized. CI of standardized estimates are in parentheses. *Model fit*: $\chi^{2}$(41) = 41.83, *p* = .43, CFI = .999, RMSEA = .016, SRMR = .043. Storage = storage capacity, Ext = externalize condition, NonExt = non-externalize condition, $D_{n}P_{m}$ with *n* = matrices test index, *m* = parcel index.

In order to test differential influences of storage capacity on Ext and NonExt without measuring error, we used a model with one single storage capacity factor (storage) and one factor for each version of matrices test (Ext and NonExt). The resulting model (Model 1, see Figure 3) fit the data very well ($\chi^{2}$(41) = 41.83, *p* = .43, CFI = .999, RMSEA = .016, SRMR = .043). Parameter estimates indicated that storage capacity had a similar relationship to both Ext (*r* = .28) and NonExt (*r* = .27). To test the equality of these correlations, we altered Model 1 by equating the parameter estimates reflecting the correlations between the two matrices tests and storage capacity. This restricted model (Model 2) was compared with Model 1. The fit of Model 2 was excellent ($\chi^{2}$(42) = 41.86, *p* = .48, CFI = 1.0, RMSEA = 0.0, SRMR = .044). Importantly, there was no significant difference in the global model fit ($\Delta \chi^{2}$(1) = 0.03, *p* > 0.05) which supports the evidence for homogeneous correlations in our bivariate correlation analyses.

## 6. Discussion

The goal of this study was to investigate the influence of storage capacity on matrix reasoning as recent studies show the significant impact of storage capacity in gF but a direct contribution of storage capacity on matrix reasoning remained unclear. Particularly, the rationale was to uncover the possible role of storage capacity in maintaining partial solutions when solving matrices test items. We therefore experimentally manipulated the demands of storing partial solutions in figural matrices tests and compared the correlations between storage capacity estimates and performance in different matrices test versions. We used a testing procedure that made it necessary to retain all information until the final response was generated (NonExt), and compared it with a procedure that provided the opportunity of using a sketchpad (Ext). We hypothesized that in the Ext condition the storage capacity would be less recruited because it was possible for participants to hold partial solutions in the external medium instead of their memory. The NonExt condition required storage of partial solutions and sub-goals which we expected to increase working memory load. Therefore, we tested whether the correlation between storage capacity and performance in the NonExt condition would be stronger than between storage capacity and performance in the Ext condition. Contrary to our expectation, results revealed that the correlation of storage capacity with performance in matrices tests did not differ between the Ext and the NonExt conditions.

This result and the fact that we replicated the substantial relationship between storage capacity and matrices test performance implies that storage capacity does not limit performance by making mistakes in buffering partial solutions. On the contrary, it might take effect earlier in the solution process. Verguts and De Boeck [18] came to a similar conclusion. They argued that “just storing partial solutions” (p. 39) cannot be the fundamental underlying mechanism that drives the relationship between working memory and matrices tests performance. Chuderski et al. [12] proposed that “the crucial cognitive mechanism underlying gF lies in storage capacity, which enables people to actively maintain distinct chunks of information and flexibly construct task-relevant bindings among them” (p. 293). However, it remains unclear how this could be demonstrated in the solving process in matrices tests. One possibility could be the building, breaking, and storage of bindings during rule induction. Carpenter et al. [22] underlined the process of pairwise comparison of matrices cells to induce the correct rule. Storage capacity could be involved in storing information within the short interval of toggling between two matrices fields and in building temporary bindings [43,44]. Alternatively, storage capacity may not be relevant for matrix reasoning because of its limit in storage but rather due to its relationship to controlled attention. According to this idea, storage capacity could be involved in rule induction, sub-goal representation and interplay with knowledge of rules from secondary memory (e.g., [25,27]).

However, our data also show that storing partial solutions is relevant for the reached performance level even though this does not explain the inter-individual differences. The small but significant decrease of performance in matrices test items from Ext to NonExt condition indicates that higher memory demands in the NonExt condition hamper the finding of a successful solution to some degree. Because this study was not designed to answer how storage capacity influences the solution process we can only speculate about the sub-processes that caused the lower performance level. One possibility is that some information is lost during generation of the complete figure. Another possibility is that the visual operations during generation of the response figure as visual operations performed during generation of the response figure (e.g., visual search or element encoding) can cause interference (e.g., [45]). However, what we can say is that these processes do not differently influence participants with different levels of matrix reasoning abilities.

One salient aspect of this study is that correlations between storage capacity and matrices test performance were lower compared to other studies. This could be for three reasons: First, usually storage capacity is compared to a latent gF factor that contains several tests. Within this study, each factor is representing matrix reasoning, which can only be regarded as one facet of gF attenuating the lower correlations found. However, the correlations between mean storage capacity and matrices tests performance are comparable to bivariate correlations of storage capacity and single matrices tests in other studies ranging from *r* = .28 to .44 [12,14,25,26].

Second, all items contained three rules as we wanted to control for confounding factors based on the amount of given visual information. Typically, items with a broader range of item difficulties are used in cognitive testing which results in larger inter-individual variance, and may also yield higher correlations. It could potentially occur that the storage of *three* partial solutions is not sufficiently working memory demanding, which could contribute to the weak effect size of externalization and the comparable contribution of storage capacity to both versions of matrices tests. However, we could exclude ceiling effects in performance which could have been indicating that the task with three rules was too simple for the participants. Moreover, the number of solved rules (28.94 of 42 for Ext and 26.21 of 42 for NonExt) indicate that items are differentiating in an average range and therefore, are sufficient for reliable correlation analyses. Items with more rules could potentially lead to floor effects which could bias the correlation analyses. Nonetheless, further studies should apply matrices tests items with a broader range of item difficulty (i.e., a broader range of number of rules). We suggest the application of experimental designs, which control for visual complexity that influence tasks demands as selective attention or goal monitoring (see Primi [33] for a good example).

Third, to control for visual salience, stimuli were created out of four element groups and four rules since there is evidence that visual salience in matrices tests can influence working memory demands [35]. Additionally, since one critical feature of gF is the flexible adaption to new problems [46], restriction of the number of rules could also lead to a restriction of variance in the performance of matrix reasoning. In other words, by excluding a number of confounding variables from our our experimental design we also excluded variables which contribute to inter-individual differences. By doing so, we restricted variance and this might have reduced the correlation between WMC and test performance. However, please note that the main rationale of this study was the comparison of the correlation of storage capacity with performance in both matrices test conditions, and we did not focus on the absolute magnitude of the correlation.

To sum up, we observed that storage capacity is related to matrix reasoning, which replicated the crucial involvement of storage capacity in gF tasks such as figural matrices tasks. But more importantly, our study showed that this relationship is not reduced when the storage demands of part solutions are removed. This suggests that the relationship between storage capacity and matrix reasoning is not caused by storage of partial solutions. There is, however, still a possibility that storage per se is relevant, e.g., if it takes effect during rule induction which cannot be decided on the basis of this experiment. Furthermore, because we did not find a direct influence of storage on reasoning performance, it is also possible that another common cause exists which influences WMC as well as reasoning. As suggested in the tradition of WMC as attention control possible candidates are resilience to distraction or the speed of disengagement. This can be investigated in the same way as we did if all demands of the reasoning task is kept constant but the demands in terms of distraction and disengagement are manipulated. In closing, two limitations of our study should be briefly discussed which are a consequence of the selected experimental approach. One is that this approach is based on the subtraction logic as originally introduced by Donders ([47] cited in [48]). It means that adding or removing storage demands does not change other processes, for example, the induction process of solving a reasoning problem. This is an assumption, and its validity cannot be directly proved. It is only possible to provide indirect evidence for this. Therefore, further studies have to tease apart the storage demands caused by the solution process from the demands of storing responses, and how both are moderated by the response format. This would address the second limitation, too. In order to control the storage demands of partial solutions, we assessed reasoning abilities by DESIGMA which has a different test format to conventional matrices tests. Conventional matrices tests present the item stem together with response options. This does not allow externalizing responses of part solutions as in the Ext condition of the present study. Nevertheless, the displayed response options with correct partial solutions may provide some memory support. Additionally, these displays can be used for a guided search for transformations of additional features which would reduce the storage demands during the solution process. The results of this study are therefore only first data on the contribution of storage demands to performances in conventional matrices tests. In future studies conventional matrices tests like the Raven APM [23] should be applied besides DESIGMA to allow inferences to matrix reasoning in general. Additionally, the inclusion of alternative tests to assess gF would ensure to evaluate the influence of storage demands not only on matrix reasoning but also on gF.

## Figures and Tables

**Table 1:** Rule types and description.

Rule Type	Description
Addition	Elements of first two cells are summed up in third cell
Subtraction	Elements of second cell are subtracted from elements in first cell and the result is shown in third cell
Overlap	Elements that are shown in first *and* second cell are presented in third cell
Addition of unique elements	Elements that are shown in first *or* second cell are presented in third cell

**Table 2:** Descriptives and correlations of fluid intelligence and storage capacity.

	M	*SD*	Min	Max	Color *k*	Shapes *k*	Ori *k*	Mean *k*	Ext	NonExt
Color *k*	3.27	0.70	1.30	4.45	**0.73**					
Shapes *k*	1.96	0.70	0.40	3.20	0.48 ***	**.72**				
Ori *k*	1.88	0.86	0.05	3.70	0.42 ***	.37 ***	**.70**			
Mean *k*	0.00	0.78	–2.07	1.36	0.81 ***	.78 ***	.76 ***	**-**		
Ext	28.94	16.17	0	48	0.18	.13	.25 *	.24 *	**.97**	
NonExt	26.21	15.79	0	48	0.16	.11	.29 *	.24 *	.81 ***	**.97**

*Note*: Color *k*, Shape *k* and Ori *k*: estimates for storage capacity for color, shape and orientation condition; Mean *k*: mean of *z*-values of Color *k*; Ext: sum of solved rules for externalize condition; NonExt: sum of solved rules for non-externalize condition; Correlations based on Pearson-Correlation; Numbers along the diagonal represent Chronbach’s α measures of reliability (note that there is no Chronbach’s α for Mean *k* since this variable is the average of values of the three conditions color, shape and orientation); * *p* < .05, *** *p* < .001.
